# Complementary and alternative medicine (CAM) use and delays in presentation and diagnosis of breast cancer patients in public hospitals in Malaysia

**DOI:** 10.1371/journal.pone.0176394

**Published:** 2017-04-27

**Authors:** Noor Mastura Mohd Mujar, Maznah Dahlui, Nor Aina Emran, Imisairi Abdul Hadi, Yan Yang Wai, Sarojah Arulanantham, Chea Chan Hooi, Nur Aishah Mohd Taib

**Affiliations:** 1 Cluster of Healthy Lifestyle, Advanced Medical and Dental Institute, University Science of Malaysia, Penang, Malaysia; 2 Department of Social & Preventive Medicine, Faculty of Medicine, University of Malaya, Kuala Lumpur, Malaysia; 3 Ministry of Health, Putrajaya, Malaysia; 4 Department of Surgery, Faculty of Medicine, University of Malaya, Kuala Lumpur, Malaysia; Sudbury Regional Hospital, CANADA

## Abstract

Complementary and alternative medicine (CAM) is widely used among the breast cancer patients in Malaysia. Delays in presentation, diagnosis and treatment have been shown to impact the disease prognosis. There is considerable use of CAM amongst breast cancer patients. CAM use has been cited as a cause of delay in diagnosis and treatments in qualitative studies, however there had not been any confirmatory study that confirms its impact on delays. The purpose of this study was to evaluate whether the use of CAM among newly diagnosed breast cancer patients was associated with delays in presentation, diagnosis or treatment of breast cancer. This multi-centre cross-sectional study evaluating the time points of the individual breast cancer patients’ journey from first visit, resolution of diagnosis and treatments was conducted in six public hospitals in Malaysia. All newly diagnosed breast cancer patients from 1st January to 31st December 2012 were recruited. Data were collected through medical records review and patient interview by using a structured questionnaire. Complementary and alternative medicine (CAM) was defined as the use of any methods and products not included in conventional allopathic medicine before commencement of treatments. Presentation delay was defined as time taken from symptom discovery to first presentation of more than 3 months. The time points were categorised to diagnosis delay was defined as time taken from first presentation to diagnosis of more than 1 month and treatment delay was defined as time taken from diagnosis to initial treatment of more than 1 month. Multiple logistic regression was used for analysis. A total number of 340 patients participated in this study. The prevalence of CAM use was 46.5% (n = 158). Malay ethnicity (OR 3.32; 95% CI: 1.85, 5.97) and not interpreting symptom as cancerous (OR 1.79; 95% CI: 1.10, 2.92) were significantly associated with CAM use. The use of CAM was associated with delays in presentation (OR 1.65; 95% CI: 1.05, 2.59), diagnosis (OR 2.42; 95% CI: 1.56, 3.77) and treatment of breast cancer (OR 1.74; 95% CI: 1.11, 2.72) on univariate analyses. However, after adjusting with other covariates, CAM use was associated with delays in presentation (OR 1.71; 95% CI: 1.05, 2.78) and diagnosis (OR 2.58; 95% CI: 1.59, 4.17) but not for treatment of breast cancer (OR 1.58; 95% CI: 0.98, 2.55). The prevalence of CAM use among the breast cancer patients was high. Women of Malay ethnicity and not interpreting symptom as cancerous were significantly associated with CAM use. The use of CAM is significantly associated with delay in presentation and resolution of diagnosis. This study suggests further evaluation of access to breast cancer care is needed as poor access may cause the use of CAM. However, since public hospitals in Malaysia are heavily subsidized and readily available to the population, CAM use may impact delays in presentation and diagnosis.

## Introduction

Complementary and alternative medicine (CAM) has become increasingly popular in Malaysia and widely used among individuals with cancer[1] especially breast cancer patients[2]. According to the National Center for Complementary and Alternative Medicine (2008), the term CAM is defined as a broad set of health care practices that are not part of a country’s own tradition and not integrated into the dominant health care system. Complementary medicine is used in addition to conventional medicine, while alternative medicine is used as replacement of conventional medicine [3].

Malaysia is a multiethnic and multicultural country consists of Malay, Chinese and Indian ethnicities that have strong traditional beliefs and practices [2]. Socio-cultural beliefs and practices influence help-seeking behavior of breast cancer patients [4,5]. Numerous studies had reported significantly high degree of CAM use in Malaysia. The prevalence of CAM use by breast cancer patients in Malaysia range from 25% to 88.3% [6–12]. High utilisation of CAM was also found in other Asian countries such as 75.0% in Indonesia [13], 67% in Korea [14], 60.9% in Thailand [15], 55.0% in Singapore[16] and 47.3% in Turkey [17].

There is no evidence claiming that complementary and alternative medicine (CAM) is more effective than conventional medicine but public opinion and interest in CAM is strong and growing. Although CAM has been reported to be commonly used among the breast cancer patients but its significance and implication to the efficacy of conventional medicine is still unclear [16,18]. The efficacy of CAM has been found to be equal to allopathic medicine in the health beliefs of Malaysian breast cancer patients and has been found to be a cause of advanced stage at presentation [19]. In Malaysia, about 30% to 56% of breast cancer patients present with advanced or stage III and IV disease [11,20,21]. Studies had found that delays is responsible for the advanced stage at diagnosis [22–24], defaulting treatment [21] and by itself is a poor prognostic factor for breast cancer survival [19,25,26].

To date, studies on CAM use amongst breast cancer patients in Malaysia are more focused on the prevalence and its associated factors [1,8,10,27]; type and pattern [9,28]; purposes [2]; knowledge [29]; and quality of life [7]. There is a scarcity of published reports on CAM use and its impact on cancer treatments. The relationship of CAM use and delays in breast cancer has not been studied extensively. Association of CAM use and delays has not been investigated.

Hence, the objective of this study was to determine the prevalence of CAM use before treatment and to evaluate whether it’s use is associated with delays in presentation, diagnosis and treatments of breast cancer. This information will assist clinicians and policy makers to formulate strategies and implement public health activities that can prevent delays in presentation, diagnosis and treatment of breast cancer in the future.

## Methods

### Design and population

This is a multi-centre cross sectional study. The study population consisted of all newly diagnosed breast cancer patients attending six public hospitals in Malaysia. Two hospitals were located in Kuala Lumpur and others in Perak, Johor, Kelantan and Sarawak. Universal sampling was conducted whereby all patients diagnosed by histopathology examination (HPE) between 1^st^ January and 31^st^ December 2012 were included. Cases were identified through the hospital registry and Surgery Out-Patient Department (SOPD) or Breast Clinic breast cancer records at each hospital. Those diagnosed with recurrent cancer and incomplete dates were excluded as this study primarily concerned with newly diagnosed breast cancer and the time evaluation. A total of 870 patients were diagnosed in the six hospitals during this time period and an effort has been made to get all the medical records. However, in situations where the medical records were not available after three requests (e.g. use by the doctors or other departments), the patients were removed from the study, giving 420 patients records that were successfully traced. Each record was examined and patients were then contacted for informed consent.

### Questionnaire and data collection

The researcher conducted an interview guided by a questionnaire that was developed from literature review. The questionnaires were in Malay and English language and were pre-tested for face and content validity amongst breast cancer survivors and breast surgeons to assess whether they met the study objectives. The questionnaire components included the use of complementary and alternative medicine (CAM), socio-demographic characteristics, medical and family history, treatment adherence and the dates of all important time points (e.g. first symptom discovery, first presentation, diagnostic resolution and initial treatment). These dates and clinical data were obtained from patient’s medical records and patient interviews. The patients were recruited in July to December 2012, hence some patients were retrospectively asked about the breast cancer journey events and patients diagnosed in July 2012 to December 2012 were followed up prospectively. All patients recruited in this study were followed up with a median of 14 months (range: 12 to 18 months) from diagnosis. The data was cross-validated between the medical records and patient interviews by the researcher to ensure for accuracy.

CAM use refers to the use of CAM before initial treatment. Question asked was “Do you use CAM? If yes, when do you use CAM, before or after treatment?”. CAM was defined as any therapy using methods and products not included in the conventional medicine (e.g. biologically based practice, mind-body medicines, whole medical system, energy medicines, manipulative and body-based practice) [21]. Delay in presentation which refers to patient delay was defined as the time taken from the first symptom discovery until first presentation at a primary care facility of more than 3 months based on several studies [23,24,30]. Delay in diagnosis which refers to patient and/or system delay was defined as the time taken from the first presentation at a primary care facility until a diagnosis resolution of more than 1 month based on several studies [31–33]. Meanwhile, delay in treatment which also refers to patient and/or system delay was defined as the time taken from the diagnosis resolution until initiation of treatment of more than 1 month based on several studies [34–36]. A family history of breast cancer was defined as having first, second or third degree relatives with cancer.

### Data analysis

All analyses were performed using SPSS version 20.0 (SPSS Inc, Chicago). Continuous data were described by median (range) whereas categorical data were described by frequencies (percentage, %). CAM use and delays in breast cancer were divided into dichotomous outcome; “Non-CAM user” or “CAM user” and “Non-delay” or “Delay”. Multivariate logistic regression was used to identify the association between CAM use and delays in presentation, diagnosis and treatment. Results were presented as adjusted odds ratio (OR), 95% confidence interval (95% CI) with a significant *p* value <0.05. Variables included in the multivariable model were CAM use, age, ethnicity, education level, marital status, household income, employment status, family history with breast cancer, breast lump, symptom interpretation, surgical services and oncology services.

Ethical approvals were obtained from the University Malaya Medical Centre (UMMC) Ethics Committee (PPUM/MDU/300/04/03), National Medical Research Registry (NMRR) ((2)dlm.KKM/NIHSEC/08/0804/P12-824) and directors from all the respective hospitals.

## Results

The 6 participating hospitals enrolled a total of 870 breast cancer patients in 2012. From this number, only 420 medical records were successfully obtained. All patient records were then reviewed, but 80 patients had been excluded due to the following reasons: recurrent breast cancer (n = 25), diagnosis of other cancer (n = 15), refusal to participate (n = 20) and incomplete dates (n = 20). Therefore, the final number analysed was 340 giving a response rate of 48.3%.

The median age was 53 years (23 to 74 years). Majority of the patients have at least a secondary education level. Most of them were unemployed with median household income of RM2, 900 (~USD 708) per month. Approximately, 17.4%, 37.6%, 33.5% and 11.5% were diagnosed at Stage I, II, III and IV respectively.

### Use of complementary and alternative medicine (CAM)

Out of 340 patients, 158 (46.5%) reported as CAM users. The median cost for CAM used was RM500 (~USD 112.40). Biological based practices (75.9%), mind-body medicines (38.6%) and whole medical system (35.4%) were the most frequent CAM used by the patients. The types of CAM used by the patients are summarized in Table 1.

**Table 1:** Types of complementary and alternative medicines (CAM) used by the breast cancer patients (n = 158).

Types of CAM		Cases (n)	Total (%)
Biological based practices			120 (75.9)
	Nutritional supplements (multivitamin)	108	
	Special diet (herbs, juices)	12	
Mind-body medicines			61 (38.6)
	Prayers	53	
	Others (meditation, tai chi, yoga, qigong)	8	
Whole medical system			56 (35.4)
	Traditional Chinese medicine	38	
	Cupping	10	
	Homeopathy	5	
	Ayurveda	3	
Energy medicines			5 (3.2)
	Ozone therapy	5	
Manipulative and body-based therapies			3 (1.9)
	Massage	3	

Total percentage may not be 100% due to the choice given for multiple responses.

Table 2 summarizes the demographic and characteristics of CAM users and non-CAM users. CAM use was seen mainly amongst the Malays, low educational status, presence of family history of breast cancer, those who did not interpret symptom as cancerous, higher cancer stage and non-adherence to treatments. CAM use was used in the 6 hospitals ranging from 42% to 75% of the patients in each hospital.

**Table 2:** Characteristic of Non CAM and CAM user among the breast cancer patients (N = 340).

Characteristic		Non-CAM user (n = 182)	CAM user (n = 158)	Adjusted OR (95% CI)	P value
Age					
	Median (range)	53 (25, 74)	53 (23,73)	0.99 (0.97, 1.01)	0.613
Study locations					
	Kuala Lumpur (1)	56 (56.0)	44 (44.0)	1.00	-
	Kuala Lumpur (2)	43 (53.8)	37 (46.3)	0.86 (0.43, 1.72)	0.672
	Perak	28 (58.3)	20 (41.7)	0.79 (0.37, 1.69)	0.544
	Johor	26 (52.0)	24 (48.0)	0.79 (0.35, 1.79)	0.586
	Kelantan	5 (25.0)	15 (75.0)	2.24 (0.64, 7.76)	0.203
	Sarawak	24 (57.1)	18 (42.9)	1.06 (0.43, 2.62)	0.884
Ethnicity					
	Chinese	71 (68.3)	33 (31.7)	1.00	-
	Malay	62 (40.3)	92 (59.7)	**3.32 (1.85, 5.97)**	<0.001
	Indian	32 (59.3)	22 (40.7)	1.37 (0.64, 2.93)	0.409
	Others	17 (60.7)	11 (39.3)	1.38 (0.53, 3.58)	0.499
Educational level					
	Tertiary	25 (51.0)	24 (49.0)	1.00	-
	Secondary	141 (54.7)	117 (45.3)	0.92 (0.46, 1.85)	0.827
	Primary	16 (48.5)	17 (51.5)	1.26 (0.44, 3.59)	0.663
Marital status					
	Married	140 (54.1)	119 (45.9)	1.00	-
	Single	42 (51.9)	39 (48.1)	1.09 (0.58, 2.06)	0.729
Household income					
	≤RM3000	129 (52.9)	115 (47.1)	1.00	-
	>RM3000	53 (55.2)	43 (44.8)	1.13 (0.65, 1.96)	0.654
Employment status					
	Employed	55 (50.5)	54 (49.5)	1.00	-
	Unemployed	127 (55.0)	104 (45.0)	0.99 (0.58, 1.68)	0.970
Family history with breast cancer					
	Yes	27 (43.5)	35 (56.5)	1.00	-
	No	155 (55.8)	123 (44.2)	0.57 (0.31, 1.05)	0.072
Symptom included breast lump					
	Yes	158 (52.7)	142 (47.3)	1.00	-
	No	24 (60.0)	16 (40.0)	0.63 (0.30, 1.32)	0.229
Interpret symptom as cancer					
	Yes	124 (57.7)	91 (42.3)	1.00	-
	No	58 (46.4)	67 (53.6)	**1.79 (1.10, 2.92)**	0.018
Stage at diagnosis					
	Stage I	33 (55.9)	26 (44.1)	1.00	-
	Stage II	74 (57.8)	54 (42.2)	0.79 (0.39, 1.58)	0.509
	Stage III	58 (50.9)	56 (49.1)	1.04 (0.50, 2.15)	0.910
	Stage IV	17 (43.6)	22 (56.4)	1.30 (0.52, 3.21)	0.565
Initial treatment					
	Adherence	155 (54.8)	128 (45.2)	1.00	-
	Non-adherence	27 (47.4)	30 (52.6)	1.34 (0.76, 2.38)	0.308

Multivariable Logistic Regression, Significant value p<0.05.

After multivariate logistic regression, ethnicity and symptom interpretation were found to be independently associated with CAM use. The odds of CAM use among Malays were 3.32 times higher (OR 3.32; 95% CI: 1.85, 5.97) than the Chinese. Meanwhile, the odds of CAM use among patients who did not interpret symptom as cancerous were 1.79 times higher (OR 1.79; 95% CI: 1.10, 2.92) than those who had interpreted symptom as cancerous. The age, study location, education level, marital status, monthly household income, employment status, family history, breast symptoms, cancer stage and treatment adherence did not independently predict CAM use among breast cancer patients in Malaysia.

### Complementary and alternative medicine (CAM) use and delays in breast cancer

Median time to presentation, diagnosis and treatment among breast cancer patients in public hospitals in Malaysia were 2.4 months, 26 days and 21 days respectively. The delay in presentation rate was 35% (n = 119), delay in diagnosis was 41.8% (n = 142) and delay in treatment was 35.3% (n = 120). Table 3 shows the association between CAM use and delays in presentation, diagnosis and treatment in breast cancer. From the analysis, it was found that CAM use was associated with delays in presentation (OR 1.65; 95% CI: 1.05, 2.59), diagnosis (OR 2.42; 95% CI: 1.56, 3.77) and treatment (OR 1.74; 95% CI: 1.11, 2.72).

**Table 3:** Univariate analysis of association between CAM use and delays in presentation, diagnosis and treatment among the breast cancer patients (N = 340).

Characteristic		Presentation		Diagnosis		Treatment	
Median (range)		2.4 months (7 days-10 years)		26 days (4 days-9.3 months)		21 days (1 day-7.2 months)	
Delays, n (%)		119 (35)		142 (41.8)		120 (35.3)	
		**Crude OR** **(95% CI)**	**P value**	**Crude OR** **(95% CI)**	**P value**	**Crude OR** **(95% CI)**	**P value**
CAM							
	Non-user	1.00	-	1.00	-	1.00	-
	User	**1.65 (1.05, 2.59)**	0.028	**2.42 (1.56, 3.77)**	<0.001	**1.74 (1.11, 2.72)**	0.015

Univariable Logistic Regression, Significant value p<0.05.

However, after adjustment with other covariates (Table 4), CAM use was only associated with delays in presentation (OR 1.71; 95% CI: 1.05, 2.78) and diagnosis (OR 2.58; 95% CI: 1.59, 4.17) but not for treatment (OR 1.58; 95% CI: 0.98, 2.55). Findings indicate that CAM users were 1.71 times higher odds to delay presentation and 2.58 times higher odds to delay diagnosis compared to non-CAM users. Besides that, symptoms without breast lumps (OR 2.17; 95% CI: 1.06, 4.42) was also the independent factors for diagnosis delay, while not having a family history of breast cancer (OR 1.81; 95% CI: 1.01, 3.26) was the independent factor for treatment delay.

**Table 4:** Multivariate analysis of association between CAM use and other characteristics with delays in presentation, diagnosis and treatment among the breast cancer patients (N = 340).

Characteristic		Delay in presentation (n = 119)		Delay in diagnosis (n = 142)		Delay in treatment (n = 120)	
		Adjusted OR (95% CI)	P value	Adjusted OR (95% CI)	P value	Adjusted OR (95% CI)	P value
CAM							
	Non-user	1.00	-	1.00	-	1.00	-
	User	**1.71 (1.05, 2.78)**	0.029	**2.58 (1.59, 4.17)**	<0.001	1.58 (0.98, 2.55)	0.058
Age							
	≤50 years	1.00	-	1.00	-	1.00	-
	>50 years	0.65 (0.39, 1.09)	0.110	1.21 (0.72, 2.02)	0.470	1.02 (0.61, 1.71)	0.919
Ethnicity							
	Chinese	1.00	-	1.00	-	1.00	-
	Malay	1.24 (0.70, 2.18)	0.449	0.90 (0.51, 1.57)	0.711	1.53 (0.86, 2.70)	0.142
	Indian	0.69 (0.32, 1.48)	0.343	1.13 (0.55, 2.33)	0.721	1.69 (0.80, 3.56)	0.164
	Others	0.81 (0.31, 2.11)	0.664	0.51 (0.18, 1.41)	0.199	0.95 (0.35, 2.54)	0.922
Educational level							
	Tertiary	1.00	-	1.00	-	1.00	-
	Secondary	1.75 (0.82, 3.72)	0.148	0.98 (0.48, 1.98)	0.959	1.03 (0.50, 2.11)	0.928
	Primary	1.88 (0.64, 5.50)	0.250	2.01 (0.71, 5.65)	0.185	1.92 (0.69, 5.34)	0.209
Marital status							
	Married	1.00	-	1.00	-	1.00	-
	Single	1.51 (0.86, 2.65)	0.146	1.27 (0.73, 2.22)	0.387	0.76 (0.43, 1.34)	0.354
Household income							
	≤RM3000	1.00	-	1.00	-	1.00	-
	>RM3000	1.56 (0.93, 2.63)	0.090	0.64 (0.37, 1.10)	0.108	1.07 (0.59, 1.70)	0.979
Employment							
	Employed	1.00	-	1.00	-	1.00	-
Unemployed		1.62 (0.93, 2.82)	0.088	0.68 (0.40, 1.17)	0.174	0.88 (0.51, 1.51)	0.648
Family history with breast cancer							
	Yes	1.00	-	1.00	-	1.00	-
	No	1.65 (0.86, 3.17)	0.126	0.80 (0.44, 1.47)	0.486	**1.81 (1.01, 3.26)**	0.049
Symptomatic breast lump							
	Yes	1.00	-	1.00	-	-Nil-	
	No	0.94 (0.45, 1.94)	0.869	**2.17 (1.06, 4.42)**	0.033		
Interpret symptom as cancer							
	Yes	1.00	-	1.00	-	-Nil-	
	No	0.77 (0.47, 1.27)	0.316	0.91 (0.56, 1.49)	0.734		
Surgical services							
Breast surgeon		-Nil-		-Nil-		1.00	-
General surgeon						1.50 (0.91, 2.48)	0.111
Oncology services							
	Available	-Nil-		-Nil-		1.00	-
Not available						0.88 (0.48, 1.62)	0.694

Multivariable Logistic Regression, Significant value p<0.05, Nil = Not included.

## Discussion

Findings of this study showed that CAM use was prevalent among the breast cancer patients in all the 6 public hospitals. However, the rate of 46.5% was lower than 51–88.3% of CAM use reported in other Malaysian studies [6–12]. The lower prevalence could be due to differences in study instruments, sample used and time point of the CAM use [29,37].

Studies on CAM use among breast cancer had found that CAM use is influenced by demographic, lifestyle and clinical factors [38]. In this study and similar to many local studies, ethnicity was found to be associated with CAM use where the Malays were observed to use CAM more than other ethnics [8,10]. Although some studies in Malaysia reported that there is no significant association between ethnicity and CAM, Malays was found to be the highest CAM user compared to Chinese and Indian [6,9,28,39]. Malays are dominated by strong community relationships where family and friends involvement greatly influence the patients’ treatment-seeking behaviour [2] which indirectly leads to CAM use. Studies in Malaysia and Singapore have found that Malay ethnicity is an independent factor of overall survival in breast cancer patients after adjustment for stage at presentation and type of treatments [5,40]. It is plausible that use of CAM may be a factor affecting the prognosis of Malay women, and this should be investigated further.

Besides that, we also observed in this study that not interpreting symptom as cancerous was significantly associated with CAM use. This highlight the importance of symptom appraisal as reported in other studies [5,41,42]. Appraisal is defined as a decision making process [43] which begins when the women discovered an abnormality in their breasts [44].

This illustrates that the symptom interpretation among the breast cancer patients in this study was poor. Other studies have also confirmed this [5,41,45]. Lack of knowledge about correct interpretation of symptoms will causes patients to have difficulty to present or decide in seeking medical attention and particularly worrisome we found from this study that CAM use was used for perceived benign conditions of the breast.

Hence, the socio-cultural influence on health behaviour of Malay women in the use of CAM as an initial help-seeking strategy for breast symptoms was compounded further by poor literacy in breast cancer symptoms. This demands an urgent call to provide culturally appropriate public health education on breast cancer symptoms and help-seeking strategies to this group of women.

Studies showed that they were influenced by family members and friends, thought that CAM works, had bad experience in hospital, financial problems, was afraid of loss of employment after the mastectomy, no time, having young children, embarrassed to see doctors, used as the last hope, easily available and affordable were the reasons for using CAM [12,44].

Early exposure to CAM causes continuation of use up to diagnosis. Fear exposure to mammographic radiation [46], fear of diagnostic test [47] and pain during biopsy procedures [48] could lead to CAM use to increase physical and emotional health [4]. Besides that, symptoms without breast lump need thorough imaging investigations and open surgical biopsy was carried out only after many non-conclusive biopsies had delayed the timing for diagnosis. To reduce diagnosis delay, image-guided biopsy is recommended where facilities and expertise are available [49,50].

Poor symptom recognition by healthcare providers may compound this further [5,51]. Findings from a study with a mixture of government and private hospitals showed timely cancer surgical services but lower achievement for radiotherapy and services [52]. Public hospitals in Malaysia provide free or highly subsidised healthcare and produces excellent maternal and child healthcare outcomes, however little is known on the efficiency in cancer care as there is no national audit on diagnostic time for cancers in Malaysia.

We did not find association between CAM use and delay in treatment. However, many studies reported association between CAM use and non-adherence to breast cancer treatments [6,7,21,52]. This suggests that the use of CAM did not interfere with the speed of provision on treatment once the patients have decided on treatments but have some influence on non-adherence to treatment.

Accessibility to health facilities in Malaysia was not a problem since the nearest health center was within 5km radius from the households [53]. Public medical care services in Malaysia is subsidized and charges a small fee [4] and was reported that 82% of breast cancer patients could access breast surgery timely [52]. These suggest that access to health care may not be the factor for delays in presentation, diagnosis, and treatment in Malaysia.

To the best of our knowledge, this is the first study to examine the impact of CAM use on delays in breast cancer. The response rate of 48.3% due to difficulty in obtaining medical records may have excluded patients who experienced delays in presentation, diagnosis or treatment thus limiting the validity of the study. A proportion of the patients were interviewed retrospectively about their journey, hence the propensity for recall bias. However, every precaution and resources were utilized to obtain record, hence a retrieval rate of 48% in busy public hospitals with limitations in manual record keeping gives a good representation. Furthermore, the study sample comprised of multi-ethnic patients in public hospitals from all regions, thus making it possible to infer the findings to all breast cancer patients in Malaysia. Linguistic and culturally appropriate health education on breast cancer symptoms and the importance of seeking early cancer diagnosis should target the people who are likely to use CAM as preferred initial treatments. In addition, the findings support that practice of CAM should be highly regulated and monitored strictly by the authorities to prevent false claims.

## Conclusion

The prevalence of CAM use among the breast cancer patients is high. Women of Malay ethnicity and not interpreting symptom as cancerous were significantly associated with CAM use. The use of CAM had significantly associated with delay in presentation and resolution of diagnosis. Difficulty in obtaining all medical records may have excluded patients who experienced delays in presentation, diagnosis or treatment but every precaution and resources were utilized to obtain record. This study suggests further evaluation of access to breast cancer care is needed as poor access may promote the use of CAM. However, since public hospitals in Malaysia are heavily subsidized and readily available to the population, CAM use may impact delays in presentation and diagnosis.

## Supporting information

S1 File QUESTIONNAIRES.docx (S1_File”). (DOCX)
