# Supplementary material for: Complementary and alternative medicine (CAM) use and delays in presentation and diagnosis of breast cancer patients in public hospitals in Malaysia
Source: PLoS One. 2017 Apr 27;12(4):e0176394. doi: 10.1371/journal.pone.0176394 (PMC5407802; doi:10.1371/journal.pone.0176394)
Supplement: S1 File — (DOCX) [file pone.0176394.s001.docx]

## APPENDIX C: CONSENT FORM

**CONSENT BY PATIENT FOR CLINICAL RESEARCH**

| I, ……………………………………………*(Name of Patient)*  *Identity* Card No ……….…………………………………  of …………………………………………………………………………………………..*(Address)*  hereby agree to take part in the clinical research (~~clinical study~~/questionnaire study/~~drug trial~~) specified below:  **Study Title:**  **PRESENTATION, DIAGNOSIS AND TREATMENT OF BREAST CANCER AMONGST WOMEN ATTENDING PUBLIC HOSPITALS IN MALAYSIA: THE TIME INTERVALS AND ASSOCIATED FACTORS TO DELAY**  whereby the nature and purpose of which has been explained to me by the investigator,  ……………………………………………… *(Name & Designation of Doctor)*  and interpreted by ................................................................... *(Name & Designation of Enumerator)*  to the best of his/her ability in …………………….…………… language/dialect.  I have been told about the nature of the clinical research in terms of methodology, possible adverse effects and complications (as per patient information sheet). After knowing and understanding all the possible advantages and disadvantages of this clinical research, I voluntarily consent of my own free will to participate in the clinical research specified above.  I understand that I can withdraw from this clinical research at any time without assigning any reason whatsoever and in such a situation shall not be denied the benefits of usual treatment by the attending doctors.  Date: ……………...……….. Signature or Thumbprint …………….……………………………  *(Patient)*  **IN PRESENCE OF THE ENUMERATOR**  I confirm that I have explained to the patient the nature and purpose of the above-mentioned clinical research.  Enumerator’s Name: ………………………………………………………    Date: ……………...……….. Signature or Thumbprint …………….……………………………  *(Enumerator)* |
| --- |

## APPENDIX D: PATIENT INFORMATION SHEET


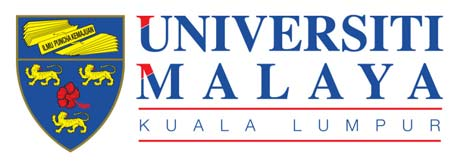


**PATIENT INFORMATION SHEET**

Please read the following information carefully, do not hesitate to discuss any questions you may have with your Doctor.

**Study Title**

**PRESENTATION, DIAGNOSIS AND TREATMENT OF BREAST CANCER AMONGST WOMEN ATTENDING PUBLIC HOSPITALS IN MALAYSIA: THE TIME INTERVALS AND ASSOCIATED FACTORS TO DELAY**

**What is the purpose of this study?**

1. To determine the time intervals between important time points in the breast cancer journey from symptom discovery to initial treatment.
2. To determine the proportion of delay in presentation, diagnosis and treatment of breast cancer patients.
3. To determine the factors associated with delay in presentation, diagnosis and treatment of breast cancer patients.
4. To verify factors associated with non-adherence to breast cancer treatments (surgery, chemotherapy, radiotherapy and hormonal therapy) amongst breast cancer patients.

**What are the procedures to be followed?**

All newly confirmed breast cancer patients will be taken as respondent. Consent form need to be filled in as permission. Purpose of study and the process of research will be explained by researcher. Respondent medical records will be reviewed and retrospectively followed up from early presentation until initial treatment. An interview session will be conducted using self-devised questionnaires to gain additional data. Respondent needs to answer and complete all questions. Trained research staff based in each hospital will conduct the interview in the language well-versed by the respondents.

**Who should I contact if I have additional questions during the course of the study?**

Investigator’s Name: **Noor Mastura Bt Mohd Mujar**

Tel: **014- 338 6411**

Email: [**mastura_mujar@yahoo.com**](mailto:mastura_mujar@yahoo.com)

## APPENDIX E: BREAST CANCER PATIENT SURVEY (BCPS)

**SOCIODEMOGRAPHIC CHARACTERISTIC INFORMATION**

Name: __________________________________________

Hosp. RN: _______________________________________

D.O.B: __________________

Age: __________ years old

Address: _________________________________________________________________

_________________________________________________________________

_________________________________________________________________

Postcode: _____________________________

Contact Number: 1) ___________________________________

2) ___________________________________

| Ethnicity | [1] Malay [2] Chinese [3] Indian [4] Others |
| --- | --- |
| Religion | [1] Islam [2] Buddhism [3] Hinduism [4] Christianity [5] Others |
| Marital status | [1] Married [2] Single [3] Divorced/Divorced [4] Unknown |
| Education | [1] Primary [2] Secondary [3] Tertiary |
| Working status | [1] Working [2] Used to work [3] Not working |
| Working sector | If working, kindly state the sector:  [1] Public [2] Private [3] Self-employed |
| Reason to left job | If used to work, kindly state the reason:  [1] Because breast cancer [2] Not because breast cancer |
| Household income (RM) | RM ______________ per month |
| Living arrangement | Currently live or stay with:  [1] Husband [2] Children  [3] Alone [4] Family member  [5] Parents [6] Friends |

**MEDICAL, OBSTRETRIC AND GINAECOLOGY INFORMATION**

| Medical History | [1] Hypertension  [2] Diabetes Mellitus  [3] Heart Disease  [4] Psychiatric Disorder  [5] Kidney Problem  [6] Others, ___________ |
| --- | --- |
| History of benign | [1] YES [2] NO |
| Parity Status | [1] Nulliparous [2] Parous |
| Pregnancies | [1] Para, _____ [2] Abortion, _____ [3] Gravida, _____ [4] Unknown |
| Age at pregnancy | [1] First child, ______ y/o [2] Last child, _____ y/o |
| Breast Feeding | [1] YES [2] NO  If Yes, what is the total duration? (inclusive of all children)  ______________ months |
| Menarche age | [1] ________ years old [2] Unknown |
| Menopausal status | [1] Post-menopausal [2] Pre-menopausal [3] Unknown  If yes, menopause age: _______ years old |
| Hormone Rep. Therapy | [1] YES [2] NO [3] Unknown |
| Alcohol | [1] YES [2] NO [3] Unknown |
| Smoke | [1] YES [2] NO [3] Unknown |
| Contraception  (Pills/Injection) | [1] YES [2] NO [3] Unknown  If yes, what type of contraceptive used:  [1] Oral [2] Injection [3] Implant |

**FAMILY HISTORY WITH BREAST CANCER**

|  |  | |
| --- | --- | --- |
| Family history with breast cancer | [1] YES [2] NO [3] Unknown | |
| If have experiences breast cancer in family members; | | |
| Total number of family members with breast cancer | _________ family members with breast cancer | |
| (Family member No.1)  Level of family member | [1] First degree  [2] Second degree | [1] Mother [2] Sister [3] Daughter  [1] Auntie [2] Niece [3] Cousin |
| Side of second degree family member | [1] Maternal [2] Paternal | |
| (Family member No.2)  Level of family member | [1] First degree  [2] Second degree | [1] Mother [2] Sister [3] Daughter  [1] Auntie [2] Niece [3] Cousin |
| Side of second degree family member | [1] Maternal [2] Paternal | |
| Total number of family members died due to breast cancer | _________ family members died due to breast cancer | |

**SYMPTOM AND PRESENTATION DETAILS**

| First breast symptom/abnormalities | [1] Painless lump [6] Changes in shape  [2] Painful lump [7] Nipple problems  [3] Swelling [8] Rashness  [4] Breast pain [9] Others  [5] Asymptomatic [10] Systemic symptom; ______________ | |
| --- | --- | --- |
| Symptoms interpretation | [1] Cancerous  [2] Non-cancerous  [3] Not sure | |
| Detection method | [1] Self detection (SD)  [2] Mammogram (MMG)  [3] Clinical breast examination (CBE) or indidental finding while admittion | |
| Performed BSE prior to presentation | [1] YES [2] NO [3] Unknown | |
| Type of primary care facility | General practitioner  [1] Private clinic (eg. GP)  [2] Public clinic (eg. KK)  Non General Practitioner  [3] OPD  [4] A&E  [5] Others: ___________ | Date: _____ / _____/ 20____  [ ] Unknown |
| Symptom Duration | ______ [1] Week [2] Month [3] Year [4] Unknown | |

**REFERRAL DETAILS**

| Referred as | [1] Urgent case  [2] Non urgent case  [3] Unknown |
| --- | --- |
| Appointment with diagnostic center | [1] Yes (went to diagnostic center **with** appointment)  [2] No (went to diagnostic center **without** appointment)  [3] Unknown |
|  | If Yes:  Appointment Date: _____ / _____/ 20____ |
|  | If No:  [1] Was given an appointment at first visit and asked to come later  Appointment Date: _____ / _____/ 20____  [2] Directly consulted at first visit without any appointment |
| Type of diagnostic center | [1] Surgery Out Patient Department (SOPD)  [2] Breast clinic |
| First consultation at diagnostic center | Date: _____ / _____/ 20____  [ ] Unknown |

**DIAGNOSTIC AND REPORT DETAILS**

| Method of  **DIAGNOSIS**  Total number of biopsy: | [1] FNAC | Date: _____ / _____/ 20____  Result: _____ / _____/ 20____ | | [ ] Cancer  [ ] Suspicious  [ ] Benign |
| --- | --- | --- | --- | --- |
|  | [2] Core Biopsy | Date: _____ / _____/ 20____  Result: _____ / _____/ 20____ | | [ ] Cancer  [ ] Suspicious  [ ] Benign |
|  | [3] Excision / Incision Biopsy | Date: _____ / _____/ 20____  Result: _____ / _____/ 20____ | | [ ] Cancer  [ ] Suspicious  [ ] Benign |
|  | [4] US Guided Biopsy | Date: _____ / _____/ 20____  Result: _____ / _____/ 20____ | | [ ] Cancer  [ ] Suspicious  [ ] Benign |
|  | [5] HWLB | Date: _____ / _____/ 20____  Result: _____ / _____/ 20____ | | [ ] Cancer  [ ] Suspicious  [ ] Benign |
|  | [6]Frozen Section | Date: _____ / _____/ 20____  Result: _____ / _____/ 20____ | | [ ] Cancer  [ ] Suspicious  [ ] Benign |
|  | *Repeat diagnosis | Date: _____ / _____/ 20____  Result: _____ / _____/ 20____ | | [ ] Cancer  [ ] Suspicious  [ ] Benign |
| Confirmed histological  **DIAGNOSIS** | Date: _____ / _____/ 20____  Location:  [1] Studied hospitals  [2] Other hospitals | | | |
| Diagnostic resolution to patient | Date: _____ / _____/ 20____  [ ] Unknown | | | |
| Breast examination  **IMAGING** | Mammogram | [1] YES [2] NO [3] Unknown  Date: _____ / _____/ 20____ | | |
|  | Ultrasound for breast | [1] YES [2] NO [3] Unknown  Date: _____ / _____/ 20____ | | |
|  | Ultrasound for axilla | [1] YES [2] NO [3] Unknown  Date: _____ / _____/ 20____ | | |
|  | Ultrasound for abdomen | [1] YES [2] NO [3] Unknown  Date: _____ / _____/ 20____ | | |
| Breast examination  **STAGING TESTS** | Chest X-Ray | [1] YES [2] NO [3] Unknown  Date: _____ / _____/ 20____ | | |
|  | Liver function test | [1] YES [2] NO [3] Unknown  Date: _____ / _____/ 20___ | | |
|  | CT Scan | [1] YES [2] NO [3] Unknown  Date: _____ / _____/ 20____ | | |
|  | Bone Scan | [1] YES [2] NO [3] Unknown  Date: _____ / _____/ 20____ | | |
| **HISTOPATHOLOGY AND STAGING DETAILS** | | | | |
| Site | [1] RIGHT | | [2] LEFT | |
| Histology type | [1] IDC  [2] DCIS  [3] ILC  [4] LCIS  [5] Paget Disease  [6] Sarcoma  [7] Other, __________________ | | [1] IDC  [2] DCIS  [3] ILC  [4] LCIS  [5] Paget Disease  [6] Sarcoma  [7] Other, __________________ | |
| Size in cm | ________cm | | ________cm | |
| Grade | [1] Grade 1 [2] Grade 2  [3] Grade 3 [4] Unknown | | [1] Grade 1 [2] Grade 2  [3] Grade 3 [4] Unknown | |
| Total of lymph node | [1] _______ LN  [2] Unknown | | [1] _______ LN  [2] Unknown | |
| Total of positive lymph node | [1] _______ LN  [2] Unknown | | [1] _______ LN  [2] Unknown | |
| ER (_______%) | [1] +VE  [2] -VE  [3] Unknown | | [1] +VE  [2] -VE  [3] Unknown | |
| PR (_______%) | [1] +VE  [2] -VE  3] Unknown | | [1] +VE  [2] -VE  [3] Unknown | |
| Clinical Staging | [1] YES  [2] NO  [3] Unknown | | | |
| Staging | **T [ ] N [ ] M [ ]** | | | |
| Primary Tumor (T) | [1] TX [2] Tis [3] T0  [4] T1 [5] T2 [6] T3  [7] T4a [8] T4b [9] T4c  [10] T4d | | [1] TX [2] Tis [3] T0  [4] T1 [5] T2 [6] T3  [7] T4a [8] T4b [9] T4c  [10] T4d | |
| Regional Lymph Nodes (N) | [1] NX [2] N1 [3] N2  [4] N3 [5] N0 | | [1] NX [2] N1 [3] N2  [4] N3 [5] N0 | |
| Distant Metastasis (M) | [1] MX [2] M0 [3] M1 | | [1] M [2] M0 [3] M1 | |
| Stage | [1] 0 [2] I [3] II  [4] III [5] IV | | [1] 0 [2] I [3] II  [4] III [5] IV | |
|  | | | | |

**BREAST CANCER TREATMENT**

**SURGERY**

| Need Surgery? | [1] YES [2] YES, but not done  [3] NO [4] Others, ___________________ | | |
| --- | --- | --- | --- |
| Surgery Date | _____ / _____/ 20____ | | |
| Site | [1] RIGHT | [2] LEFT | |
| Location of surgery | [1] UMMC [2] HKL [3] HSIJB  [4] HRPZII [5] HRPB [6] HUS  [7] Other hospital: __________________________________ | | |
| Surgical Procedure | [1] Mastectomy without recon (MAC)  [2] Mastectomy with recon  [3] Breast conserving surgery (BCS)  [4] Others  Surgical procedure: _______________________________________ | | |
| Have patient ever postponed the surgery? | [1] YES [2] NO [3] UNKNOWN  Actual surgery date (before postponed): _____ / _____/ 20____ | | |
| Treatment status | [1] Timely done  [2] Transferred to another hospital for treatment | | Completed |
|  | [3] Defaulted  [4] Lost to follow-up  [5]Others, _________________________________ | | Incomplete |
| Conclusion | [1] Adherence to surgery  [2] Non-adherence to surgery | | |

**CHEMOTHERAPY**

| Need Chemotherapy? | [1] YES [2] YES, but not done  [3] NO [4] Others, ___________________ | |
| --- | --- | --- |
| Location of chemotherapy | [1] UMMC [2] HKL [3] HSIJB  [4] HRPZII [5] HRPB [6] HUS  [7] Other hospital: __________________________________ | |
| Number of cycle | _________ cycles  Completed cycle : __________ cycles  Incomplete cycle : __________ cycles | |
| Start date | _____ / _____/ 20____ [2] Unknown | |
| End date | _____ / _____/ 20____ [2] Unknown | |
| Have patient ever postponed the chemotherapy? | [1] YES [2] NO [3] UNKNOWN  Actual chemotherapy date (before postponed): _____ / _____/ 20____ | |
| Treatment status | [1] Timely done  [2] Incomplete due to medical indications  [3] Transferred to another hospital for treatment | Completed |
|  | [4] Defaulted  [5] Incomplete cycles due to non-medical reasons | Incomplete |
| Conclusion | [1] Adherence to chemotherapy  [2] Non-adherence to chemotherapy | |

**RADIOTHERAPY**

| Need Radiotherapy? | [1] YES [2] YES, but not done  [3] NO [4] Others, ___________________ | |
| --- | --- | --- |
| Location of radiotherapy | [1] UMMC [2] HKL [3] HSIJB  [4] HRPZII [5] HRPB [6] HUS  [7] Other hospital: __________________________________ | |
| Radiotherapy site | [1] Breast [2] Supraclavicular fossa [3] Boost to tumor bed  [4] Chest wall [5] Axilla [6] Others, __________ | |
| Start date | _____ / _____/ 20____ [2] Unknown | |
| End date | _____ / _____/ 20____ [2] Unknown | |
| Have patient ever postponed the radiotherapy? | [1] YES [2] NO [3] UNKNOWN  Actual radiotherapy date (before postponed): _____ / _____/ 20____ | |
| Treatment status | [1] Timely done  [2] Incomplete due to medical indications  [3] Transferred to another hospital for treatment | Completed |
|  | [4] Defaulted  [5] Incomplete due to non-medical reasons | Incomplete |
| Conclusion | [1] Adherence to radiotherapy  [2] Non-adherence to radiotherapy | |

**HORMONAL THERAPY**

| Need hormonal therapy? | [1] YES [2] YES, but not done  [3] NO [4] Others, ___________________ | | |
| --- | --- | --- | --- |
| Location of hormonal therapy | [1] UMMC [2] HKL [3] HSIJB  [4] HRPZII [5] HRPB [6] HUS  [7] Other hospital: __________________________________ | | |
| Hormonal therapy type | [1] Tamoxifen [2] Aromasin [3] Arimidex  [4] Femara [5] Others, __________ | | |
| Start date | _____ / _____/ 20____ [2] Unknown | | |
| Changes in hormonal therapy | [1] Changed hormonal therapy  Date: _____ / _____/ 20____  Reason: _________________________ | [2] Stopped hormonal therapy  Date: _____ / _____/ 20____  Reason: __________________________ | |
| Treatment status | [1] Continue as recommended  [2] Stopped due to medical indications  [3] Transferred to another hospital for treatment | | Continue |
|  | [4] Defaulted to consume  [5] Defaulted to continue | | Incomplete |
| Conclusion | [1] Adherence to hormonal therapy  [2] Non-adherence to hormonal therapy | | |

**COMPLEMENTARY & ALTERNATIVE MEDICINE (CAM)**

| Have you ever used/practices any forms of CAM? | [ ] YES [ ] NO [ ] UNKNOWN |
| --- | --- |
| When you used CAM? | [ ] Before treatment  [ ] After treatment |
| Total cost of CAM? | RM_________________ |
| Types of CAM used?  (can answer more than one) | 1. Biological based practices  [ ] Nutritional supplements (multivitamin)  [ ] Special diet (herbs, juices)  2. Mind –body medicines  [ ] Prayers  [ ] Others (meditation, tai-chi, yoga, qigong)  3. Whole medical system  [ ] Traditional Chinese medicine  [ ] Cupping  [ ] Homeopathy  [ ] Ayurveda  4. Energy medicines  [ ] Ozone therapy  5. Manipulative and body-based therapies  [ ] Massage |

**SOURCE OF INFORMATION**

| Who is the first person to be informed about the symptom? | [1] Not disclose  [2] Spouse  [3] Children  [4] Parent  [5] Siblings  [6] Relatives  [7] Friends |
| --- | --- |
| Who is the first person to be informed about the diagnosis? | [1] Not disclose  [2] Spouse  [3] Children  [4] Parent  [5] Siblings  [6] Relatives  [7] Friends |
| Who made the treatment decision? | [1] Self decision  [2] Spouse  [3] Children  [4] Parent  [5] Siblings  [6] Relatives  [7] Friends |
| Mode of payment | [1] Payment made by patient  [2] Payment made by employer (both public and private sector)  [3] Payment made by personal insurance  [4] Unknown |
| Estimated total cost of treatment | RM: ________________ |
| The three (3) most important source of information during breast cancer treatment? | [ ] Doctors  [ ] Nurses  [ ] Family members  [ ] Women with breast cancer  [ ] Magazines/ Books on breast cancer  [ ] Newspapers  [ ] Internet  [ ] Friends  [ ] Television  [ ] Others |
